# Perspectives on Odor-Based Control of Tsetse Flies in Africa

**DOI:** 10.3389/fphys.2022.831618

**Published:** 2022-02-18

**Authors:** Paul O. Mireji, Clarence M. Mang’era, Billiah K. Bwana, Ahmed Hassanali

**Affiliations:** ^1^Biotechnology Research Institute, Kenya Agricultural and Livestock Research Organization, Kikuyu, Kenya; ^2^Department of Biochemistry and Molecular Biology, Egerton University, Njoro, Kenya; ^3^Department of Biological Sciences, University of Embu, Embu, Kenya; ^4^Department of Chemistry, School of Pure and Applied Sciences, Kenyatta University, Nairobi, Kenya

**Keywords:** tsetse fly (*Glossina* spp.), attractants and repellents, odor, ecofriendly, vector-control strategy

## Abstract

Tsetse-transmitted trypanosomiases are among the most neglected tropical diseases in sub-Sahara Africa. Although all tsetse species are susceptible to trypanosome infections, their differential attraction/feeding preferences for different wildlife, domestic animals, and/or humans constitute critical determinants of trypanosomes species they predominantly transmit. Artificial bait technologies, based on long-range tsetse olfactory responses to natural cues emitted by preferred hosts and blends of synthetic versions that mimic these cues, have successfully been applied in attractant-odor-based (“pull” tactic) reduction of field populations of some tsetse species. Olfactory attribute associated with active avoidance of tsetse-refractory non-hosts has similarly been exploited in design of repellent-odor-based (“push” tactic) protection of livestock. These tactics have opened possibility of spatially strategic deployment of the two sets of odor baits in “push-pull” tactics. Possibility of developing blends with enhanced attraction and repellence compared with those associated with savannah tsetse fly hosts and non-hosts, respectively, have been explored, where structure activity and blends of different components generated two novel blends. The studies evaluated structure activity and blends of different components. One based on attractive constituents associated with buffalo (*Syncerus caffer*) comprised of ε-nonalactone, nonanoic acid, 2-nonanone (in 1:3:2 proportion) delivered together with acetone, which showed significantly better attractancy on savannah tsetse fly than the standard blend comprised of 3-propylphenol, octenol, p-cresol, and acetone (POCA). The other blend comprised of δ-nonalactone, heptanoic acid, 4-methylguaiacol and geranylacetone (in 6:4:2:1 proportion) was significantly more repellent than previously characterized blend based on tsetse fly refractory waterbuck (*Kobus defassa*) constituents (δ-octalactone, pentanoic acid, guaiacol and geranylacetone). So far, no effective attractants or repellents of riverine tsetse fly species have been characterized. Optimized attractant and repellent blends for savannah tsetse flies lay down useful groundwork for future development of the “push-pull” deployment tactic for area-wide control of tsetse flies. Better understanding of the physiological, cellular, and molecular basis of response in the tsetse fly to odors can potentially augment the current tsetse fly-control interventions.

## Introduction

Different groups of tsetse fly species are distributed in almost all of sub-Sahara Africa and transmit Human African Trypanosomiasis (HAT) and Animal African Trypanosomiasis (AAT) causative trypanosomes (Simarro et al., 2010; Cecchi et al., 2015), infesting ten million-square kilometers in 36 countries. The flies are obligate blood-sucking Diptera in the family *Glossinidae* and genus *Glossina*. The 22 species in this genus belong to *Fusca, Palpalis*, and *Morsitans* groups. Thirteen species, mainly inhabiting rainforest, constitute the *Fusca*, while five species, predominantly inhabiting rainforests and savannah woodlands, form the *Papalis* group. The *Morsitans* group consists of five species of which *Glossina morsitans morsitans*, *Glossina pallidipes*, *Glossina swynnertoni* and *Glossina longipalpis* (Colvin and Gibson, 1992) are restricted to the savannah, while *Glossina austeni* occupy coastal forests. Within this group, *G. m. morsitans* and *G. pallidipes* are most widespread and common vectors of livestock trypanosomosis (Jordan, 1986).

The HAT constitutes one of the most neglected tropical diseases (NTDs) with devastating health and economic consequences in sub-Sahara Africa, with most recent epidemic reported between 1990 and 2015 (Brun et al., 2010; Aksoy et al., 2017). Likewise, AAT is rampant in livestock inhabiting tsetse fly-infested areas throughout the continent. The AAT causes death of about three million cattle each year, inflicting a direct loss of between US$1.0 and 1.2 billion every year from bovine trypanosomosis alone in sub-Sahara Africa (FAO, 2019). Brought largely under control in 1960s, HAT re-emerged and resurged to epidemic proportions by the end of the 20th century due to decreased control and surveillance activities. Concerted and collaborative control efforts over the last decade reversed the epidemic trend of the human disease associated with *Trypanosoma gambiense*, reducing the cases to just 6,228 by 2013 (Franco et al., 2014). Informed by the progress in HAT control, the WHO Strategic and Technical Advisory Group for NTDs declared a target to eliminate *T. gambiense* HAT as a public health problem by 2020 (WHO, 2012). However, alarming numbers are still being reported across the African continent (Tong et al., 2011; Rimoin and Hotez, 2013; WHO, 2021). Chemotherapeutic control has not been sustainable due to widespread and increasing resistance of trypanosomes to existing drugs (Barrett et al., 2011; Chitanga et al., 2011; Kulohoma et al., 2020) and high cost and sporadic availability of drugs in endemic areas (Muhanguzi et al., 2015). Chemotherapy has also been jeopardized by presence of wildlife trypanosome where these wild hosts reservoirs maintain and promote infections at the livestock–wildlife interface (Kasozi et al., 2021). Mammalian vaccines against HAT are still in their early stages of discovery due to the complex mechanism of antigenic variations associated with the trypanosome parasite (Onyilagha and Uzonna, 2019; Autheman et al., 2021).

## Control of Tsetse Fly Populations

The long-lasting adult stage of tsetse fly and exclusive hematophagy in both sexes (Attardo et al., 2019) have led to widespread epidemic of the disease, forcing farmers and herdsmen to either abandon wide areas of land across Africa, covering different ecological habitats, or to maintain their herd under regular chemotherapy (Giordani et al., 2016). Rearing of trypanotolerant livestock has been tried with minimal success (Orenge et al., 2012). Tsetse fly control is a promising means of disease control and appears to constitute the cornerstone in the disease suppression (Bouyer et al., 2007; Van den Bossche and Delespaux, 2011). Since tsetse flies are viviparous and pupal developments occur below the surface, only the adult phase of the fly is accessible to control using available tools and initiatives. The control was initially effected by elimination of wildlife hosts of tsetse flies and destruction of tsetse fly-preferred habitats (Hocking et al., 1963). Although effective, these strategies were later abandoned due to environmental concerns (Dransfield et al., 1991).

The advent of modern insecticides ushered in a massive eradication campaign of the Savannah tsetse fly species. Discovery of toxicological properties of dichlorodiphenyltrichloroethane (DDT) to tsetse flies boosted the eradication campaign, making tsetse fly control increasingly dependent on insecticides (Kabayo, 2002). DDT was considered cheap, persistent, and highly effective against tsetse flies. Several other insecticides were subsequently discovered, evaluated, and adopted in tsetse fly-control programs to various extents (Kabayo, 2002). The control was by aerial and ground insecticide spraying (Shereni, 1990). Duration of apparent reluctance to accept and expand the role of insecticides was later experienced since insecticides appeared to provide only a temporary solution to a permanent problem, manifested by tsetse fly re-infestation of cleared areas due to temporal degradation of insecticide toxicity, among other factors (Allsopp, 1984). These shortcomings, coupled with environmental hazards posed by the insecticides, shifted the control emphasis in favor of environment-friendly methods that include use of (1) Sequential Aerosol Technique (SAT) spraying of ultra-low volume non-residual insecticides that targeted adult flies in direct contact with spray mist, and subsequently emerging adults (Alsopp and Hursey, 2004; Perkins and Ramberg, 2004), successfully applied in *Glossina morsitans centralis* eradication campaign in Okavango Delta, Botswana (Kgori et al., 2006), (2) Sterile Insect Technique, (SIT) typically employed as part of area-wide integrated pest management (AW-IPM) (Klassen, 2005) in combination with other control methods as witnessed in the 4-year SIT campaign on the island of Zanzibar that achieved a historic breakthrough success in complete tsetse fly elimination from the island (Vreysen et al., 2000), (3) live animal bait technologies (Leak, 1998) and (4) visual traps and targets with effective attractants (Brightwell et al., 1991; Vale, 1993; Green, 1994). The live animal bait technologies (insecticide application to cattle) for tsetse fly control were demonstrated in southern Africa in the mid-1980s, but seemed of limited use where the intention was to remove tsetse flies from the vast invasion source or where cattle were absent or could not be introduced (Colvin and Gibson, 1992; Vreysen et al., 2013).

## Development of Tsetse Fly Visual and Olfactory Attractants

Insects use visual, olfactory, gustatory, tactile stimuli, humidity, and light intensity sensory cues to discriminate hosts from non-hosts from varying distances (Bruce et al., 2005). Color, shape and movement visual cues have close-range (<10 m) importance among tsetse flies, where they assist the flies to land on the host (Leak, 1998; Gikonyo et al., 2000). Savannah tsetse flies, in general, are visually attracted by blue color and tend to land on black surfaces (Green, 1994), with different patterns of arrangement of blue/black in traps/targets corresponding to different tsetse fly species. For example, blue centers and blacks on flanking sides from each side of the target are optimized for *Glossina palpalis palpalis* as opposed to black centers and blue sides typically preferred by *G*. *pallidipes* and *G. m. morsitans.*

Efficacy of the visual attractants is enhanced by integrating tsetse fly olfactory attractant components into the devices. The *G. m. morsitans* and *G. pallidipes* are preferentially attracted to volatiles from ungulates and other large mammals, with buffalo (*Syncerus caffer*) and cattle (*Bos taurus*) as most attractive (Grootenhuis and Olubayo, 1993; Moloo, 1993). This attraction is attributed to specific host chemical components in volatiles emanating from the hosts (Moloo, 1993). Initially, trapping of breath volatiles and their (breath volatile) chemical analysis (with gas chromatography linked with mass spectrometer and electro-antennographic measurements), followed by laboratory and field assays identified acetone, 2-butanone and 1-octen-3-ol key constituents in the natural mammalian host (buffalo and cattle) odors as attractive to tsetse flies (Hall et al., 1984). Deployment of blends consisting of these compounds significantly improved field performance (catch) of traps on the tsetse flies. However, the attraction efficiency fell below (<20%) that of natural preferred host animal (e.g., buffalo, cattle) (Vale, 1977). This pointed to a possible involvement of additional kairomones from these natural tsetse fly hosts. Further assessments revealed better attraction of the flies by the buffalo urine than blends associated with host breath, especially when fermented for a couple of days, suggesting that the urine harbored other components responsible for the enhanced attractance of the natural hosts (Owaga et al., 1988). Isolation and characterization of the fermented urine identified a phenolic blend (phenol, 3- and 4-cresols, 3- and 4-ethylphenols and 3- and 4-*n*-propylphenols) and specific combination of 4-cresol and 3-*n*-propyl phenol, as key attractants of the flies in the urine (Hassanali et al., 1986; Owaga et al., 1988). Further studies established that the release of phenols (4-methylphenol and 3-n-propylphenol) from pro-attractant derivatives was facilitated by microbiota (Poldy, 2020). These components were subsequently formulated into the 1:4:8 blend of the 3-*n*-propylphenol (P), octanol (O), and *p*-cresol (C), together with separately released acetone (A) (collectively referred to as POCA) that had enhanced attraction to *G*. *m*. *morsitans* or *G*. *pallidipes* (Owaga et al., 1988; IAEA, 2003; Rayaisse et al., 2010). However, POCA shows about 25% attraction relative to odors of natural hosts (cattle or buffalo) (Vale, 1977). Recent structure activity studies have identified attraction property of ε-nonalactone to *G*. *m*. *morsitans* and *G*. *pallidipes* (Wachira et al., 2016). In a follow-up blending studies, combination of ε-nonalactone with nonanoic acid and 2-nonanone in the 1:3:2 ratio at optimized release rate of 13.71 mg/h was about two-folds more attractive to *G*. *pallidipes* than POCA (Wachira et al., 2021) and thus constitutes a Novel Attractant Blend (NAB) tool against most savannah species tsetse flies. Performance of NAB relative to odor from the natural hosts remains to be determined. A more significant “pull” of the tsetse flies than the natural host can potentially diminish transmission of trypanosomiases by diverting the flies to dead-end traps/targets.

No optimal attractant has been formulated against *G. austeni* or *Palpalis* (riverine) species, including *G. f. fuscipes* and *Fusca* groups, and thus continue to be an active area of research, primarily focused on odors from their natural preferred hosts, including humans, cattle, pigs or monitor lizards (Mohamed-Ahmed, 1998; Omolo et al., 2009; Rayaisse et al., 2010). Fungus-based dissemination technique has been proposed for potential integration with the odor-baited trap technology where tsetse flies are trapped in a fungus-contaminated device mounted on a trap, which contaminates the flies on contact and then permits them to “escape” for subsequent contamination of wider tsetse fly populations (Maniania, 1998). This approach is still at the conceptual stage and has not been integrated into tsetse fly-control programs. Relative low cost, community acceptability, ability to stem tsetse fly re-invasion from adjacent areas (Allsopp, 1984; Mangwiro et al., 1999), high specificity and minimal environmental contamination made the odor-baited traps and targets the preferred technology for the control of tsetse flies in sub-Saharan Africa (Kabayo, 2002).

## Development of Tsetse Fly Repellents

Tsetse fly species are differentially attracted to and feeding on specific vertebrates irrespective of their relative abundance (Grootenhuis and Olubayo, 1993; Moloo, 1993). In Lambwe Valley, Kenya, *G. pallidipes* derived over 80% of their blood meals from bushbuck (*Tragelaphus sylvaticus*), buffalo (*S. caffer*), and bushpig (*Potamochoerus larvatus*), but hardly any from numerically common species, such as oribi (*Ourebia ourebi*), impala (*Aepyceros melampus*), waterbuck (*K. defassa*) and hartebeest (*Alcelaphus buselaphus*) (Turner, 1987). Likewise, in Luangwa Valley, Zambia, *G. m. morsitans* and *G. m. submorsitans* preferentially (>90%) fed on warthog (*Phacochoerus africanus)*, hippopotamus (*Hippopotamus amphibius*), bushpig, cattle (*Bos taurus*), and buffalo but not on black rhinos (*Diceros bicornis*), zebra (*Equus quagga*), giraffe (*Giraffa camelopardalis peralta*), waterbuck and impala (Clausen et al., 1998). In enclosure experiments with ox (*Bos indicus*), buffalo, eland (*Taurotragus oryx*) and waterbuck, very few tsetse flies associated with waterbuck with none engorging on this bovid (Grootenhuis and Olubayo, 1993). A series of hypotheses were projected to account for this phenomenon, including differences in the host body size/mass factor known to affect close-range attraction and landing behavior of tsetse flies. However, these factors could not account for differences between animals of roughly the same size. Suitability of host blood to the tsetse fly survival was also explored as an alternative hypothesis. However, survival and reproductive performance of *G. m. morsitans* were not influenced by *in vitro* blood feeding from preferred (buffalo, cattle, warthog) versus tsetse fly refractory bovids (waterbuck, oryx) (Moloo et al., 1988). In addition, hypothesis based on differential grooming behavior (tail flicking, skin twitching, kicking and/or stamping) between preferred and non-preferred hosts was interrogated but rejected due to the absence of negative correlation between grooming behavior of some hosts and preferential feeding (Clausen et al., 1998).

These findings led to the suggestion that refractory animals are either ignored because of emission of sub-optimal levels of key attractive semiochemicals (kairomones) or are actively avoided due to the presence of close range allomones that repel and/or deter tsetse flies from feeding (Gikonyo et al., 2000). Comparison of the behavior of teneral *G. m. morsitans* confined in small experimental cages on waterbuck and ox, and on feeding membranes with and without smears of waterbuck sebum suggested the presence of volatile and short-range or contact allomones from the waterbuck (Gikonyo et al., 2000). Examination of odor profiles of three bovids (buffalo, ox and ox) by gas chromatography-linked mass spectrometry (GC-MS) and gas chromatography-linked electroantennography (GC-EAD) revealed 15 waterbuck-specific compounds (Gikonyo et al., 2002). These included straight chain carboxylic acids (C5-C10), 2-alkanones (C8-C12 homologs and geranyl acetone), phenols (guaiacol and carvacrol) and δ-octalactone. Subsequent laboratory two-choice wind tunnel assays with *G. m. morsitans* showed a pattern of responses to synthetic blends of these compounds that suggested avoidance behavior that significantly differed from their responses to attractive blends associated with odors of preferred hosts (Gikonyo et al., 2003). Field evaluation of effects of different blends of these compounds on catches of *G. pallidipes* in attractant-baited Ngu traps showed that each class of constituents (carboxylic acids, ketones, phenols, and δ-octalactone) significantly contributed to the reduction of catches of the flies (Bett et al., 2015). However, large variations in intrinsic individual activity to *G. pallidipes* within each multicomponent group (carboxacids, ketones, and phenols) were observed. Among the carboxylic acids, the lower (C5–C7) homologs were repellent, while their higher (C8–C10) homologs were not. Among the ketones, higher molecular weight compounds (C11, C12 homologs and geranyl acetone) were significantly more repellent than their lower molecular weight (C8–C10) homologs. Of the two phenols, guaiacol was more repellent than carvacrol. Guaiacol was previously shown to be a mild tsetse fly repellent (Torr et al., 1996), and in a structure-activity study with different analogs of this phenol, replacement of H (hydrogen) with the CH_3_ (methyl) group significantly increased repellency to tsetse flies (Saini and Hassanali, 2007). Blending each class of constituents (acids, ketones, phenols, and δ-octalactone) revealed incremental contribution to the repellency of the waterbuck odor on *Glossina pallidipes* (Bett et al., 2015) with blends comprising of guaiacol, geranylacetone, pentanoic acid and δ-octalactone, constituting an optimal blend that provided significant (>80%) protection to livestock from trypanosome infection (Saini et al., 2017). Follow-up studies on effects of structural variants (analogs) of δ-octalactone on olfactory responses of *G. pallidipes* and *G. m. morsitans* established that increasing the hydrophobic chain length from C3 (δ-octalactone) to C4 (δ-nonalactone) enhanced repellency to both species, while increasing the ring size from six (δ-octalactone) to seven members (ε-nonalactone) transformed the activity of the resulting analog from repellence to attractance (Wachira et al., 2016). Moreover, combinations of specific repellent compounds (δ-nonalactone, heptanoic acid, 4-ethyl guaiacol and geranyl acetone in 6:4:2:1 proportions) substantially increased the repellence of the resulting novel blend (Wachira et al., 2016), with 95% repellency against *G. pallidipes*, relative to un-baited control traps (Wachira et al., 2020), indicating incremental effect on savannah tsetse flies relative to previously established repellents (Bett et al., 2015; Saini et al., 2017). Attractant and repellent constituents, analogs, and blends effective against the *Palpalis* (riverine) tsetse fly species, including *G. f. fuscipe*s remain to be established.

## Future Perspectives

The results outlined above lay down groundwork for the development of “push-pull” approach for effective control of tsetse flies. However, concurrent deployment of bait and repellent technologies has demonstrated the importance of adequate spatial separation of the two to avoid “confusing” the tsetse flies that encounter both downstream attractive and repellent plumes. Thus, allowing animals with repellent-control release devices to get closer to traps/targets with attractant-control release devices would reduce the kill rate for the traps/targets through a mix-up in chemical cues and expulsion of tsetse flies from the circle of attraction. Models on spatial dimension of an emotaxis-modulated host location behavior of the flies should be developed to facilitate minimal concurrent encounters of the attractant and repellent plumes under conditions of variable wind direction. The models will optimize strategic spatial deployment of traps/targets baited with a selected attractant blend and vertebrate hosts carrying controlled-release repellent devices of selected repellent blends in large-scale field control of target tsetse flies.

Receptors, neurons, and circuits (including odorant receptors, gustatory receptors, ligand-gated ionotropic receptors, odorant-binding proteins, chemosensory proteins, sensory neuron membrane proteins, and CD36-like pheromone sensors) that higher flies use to decode ecological odors and triggers for odorant-specific behavioral responses are well documented (Benton et al., 2007, 2009; Su et al., 2009; Carey and Carlson, 2011; Olivier et al., 2011; He and Carlson, 2017; Sun et al., 2018). Limited information on chemosensory/cellular processes underpinning responses of tsetse flies to odors points to their to potential application in tsetse fly control (Den Otter and Van der Goes van Naters, 1993; Saini et al., 1996). Recent availability of genomes of a suite of tsetse fly species (*G. m. morsitans*, *Glossina palpalis gambiensis*, *G. f. fuscipes*, *G. pallidipes*, *Glossina brevipalpis*, and *G. austeni*) (Attardo et al., 2019; International Glossina Genome Initiative [IGGI], 2014) has facilitated annotations (structural, expansions, and expression profiles) of chemosensory genes in tsetse flies (Liu et al., 2010, 2012; Obiero et al., 2014; Macharia et al., 2016; Kabaka et al., 2020). The annotations revealed significant reduction in some of the chemosensory genes in tsetse flies, relative to fruit flies (*Drosophila Melanogaster*) and malaria mosquitoes (*Anopheles gambiae*), putatively attributed to relatively less complex ecology, restriction in food preferences by obligate hematophagy and a narrower host range in tsetse flies. Physiological and molecular functional assessment of *G. m. morsitans* antennae identified seven functional classes of olfactory sensilla responsive to established tsetse flies attractants/repellents, carbon dioxide, vertebrate odorants, among others (Soni et al., 2019), Or35 as responsive to 1-hexen-3-ol and Or9 to acetone, 2-butanone, and 2-propanol (Chahda et al., 2019). Such information can potentially augment existing odor-based control strategies by facilitating identification of functional novel attractant/repellent odorant(s) and design of more effective field intervention strategies. However, these findings are still many experiments away (e.g., field assessment, validations, and optimization) from adoption into integrated routine tsetse fly control operations.

## Author Contributions

PM conceived the idea, coordinated, and oversaw the study. CM, BB, and PM developed the manuscript. CM, BB, PM, and AH reviewed the manuscript. All authors read and approved the final manuscript.

## Conflict of Interest

The authors declare that the research was conducted in the absence of any commercial or financial relationships that could be construed as a potential conflict of interest.

## Publisher’s Note

All claims expressed in this article are solely those of the authors and do not necessarily represent those of their affiliated organizations, or those of the publisher, the editors and the reviewers. Any product that may be evaluated in this article, or claim that may be made by its manufacturer, is not guaranteed or endorsed by the publisher.
